# Three-dimensional modelling of the choroidal angioarchitecture in a multi-ethnic Asian population

**DOI:** 10.1038/s41598-022-07510-y

**Published:** 2022-03-09

**Authors:** Kai Xiong Cheong, Kelvin Yi Chong Teo, Yih Chung Tham, Ralene Sim, Shivani Majithia, Jia Min Lee, Anna Cheng Sim Tan, Ching-Yu Cheng, Chui Ming Gemmy Cheung, Rupesh Agrawal

**Affiliations:** 1 Singapore Eye Research Institute, Singapore National Eye Centre, Singapore, Singapore; 2 Ophthalmology and Visual Sciences Academic Clinical Program (Eye ACP), Duke-NUS Medical School, Singapore, Singapore; 3 National Healthcare Group Eye Institute, Tan Tock Seng Hospital, 11 Jalan Tan Tock Seng, Singapore, 308433 Singapore; 4 Lee Kong Chian School of Medicine, Singapore, Singapore; 5 Moorfields Eye Hospital, NHS Foundation Trust, London, UK

**Keywords:** Biomarkers, Medical research

## Abstract

This study aimed to describe the topographic variation of the macula’s choroidal angioarchitecture using three-dimensional (3D) choroidal vascularity index (CVI) of healthy eyes from an Asian population and to investigate the associations of CVI. 50 participants were recruited via stratified randomisation based on subfoveal choroidal thickness from the Singapore Epidemiology of Eye Diseases Study. Macular volume scans were acquired using spectral-domain optical coherence tomography with enhanced depth imaging. CVI was assessed based on B-scan binarisation and choroid segmentation. The 3D CVI of the whole, superior, central, and inferior macula were 62.92 ± 1.57%, 62.75 ± 1.93%, 63.35 ± 1.72%, and 62.66 ± 1.70%, respectively, pairwise comparisons P all > 0.05). 3D CVI (Whole Macula) and 2D CVI (Subfoveal) were associated only with each other and not with other ocular and systemic factors. 2D CVI (Subfoveal) had a moderate agreement with 3D CVI (Central Macula) [intraclass corelation coefficient (ICC) = 0.719], and had poorer agreement with 3D CVI of the whole macula, superior, and inferior macula (ICC = 0.591, 0.483, and 0.394, respectively). Scanning volume did not influence 3D CVI measurements. In conclusion, 3D CVI demonstrated no significant topographic variation. CVI was not correlated with demographic or ocular structural features. 2D CVI of the fovea is partially representative of 3D CVI of the macula.

## Introduction

The study of the choroid provides insights into the normal physiology of the eye and the pathogenesis of chorioretinal diseases^1,2^. The choroid’s physiological roles include metabolic support to the retina and retinal pigment epithelium (RPE), blood supply to the prelaminar region of the optic nerve, and being a light and heat sink^3^. The choroid also plays important roles in chorioretinal diseases, including age-related macular degeneration^4,5^, polypoidal choroidal vasculopathy^6^, central serous chorioretinopathy^7^, diabetic retinopathy^8,9^, high myopia^10,11^, intraocular inflammatory disease^12,13^, and it may serve as a site for tumour and infection metastasis^1^.

There is considerable interest in describing the choroid using quantifiable parameters, with the aims of establishing normative reference values for ocular and systemic health, diagnosing and prognosticating disease, and monitoring treatment progress. One example is choroidal thickness (CT). However, many systemic and ocular factors can influence CT^14–16^. These include axial length (AL), spherical equivalent (SE), diurnal variation, chorioretinal diseases, age, ethnicity, and conditions that affect the cardiovascular system. Furthermore, CT reflects the entire layer of intervening tissue from the choroid-scleral interface to the RPE posterior edge and does not distinguish between stromal and luminal components^15^.

Choroidal vascularity index (CVI), which is defined as the ratio of luminal area to total choroidal area, is used as another biomarker to evaluate the choroid’s vascular status^1,2^. CVI has the advantage over CT of evaluating the choroid’s sub-compartments^1,2^. CVI is also a more robust and stable biomarker compared with CT and is more resistant to the influence of ocular and systemic factors^1,2,17,18^.

In most studies, the assessment of the choroid’s vascular status is based on two-dimensional (2D) CVI measurements of a single optical coherence tomography (OCT) B-scan passing through the fovea and/or of selected B-scans in the posterior pole^4–13^. For example, the 2D CVI of the choroid in the foveal scan of healthy individuals is reported to be 65.61% in one study^17^. There is a potential for measurement error due to the limited area of the assessed region, arbitrary selection of a single or few OCT B-scans, and projection artifacts^19^. A better strategy of studying the choroid may involve assessing the three-dimensional (3D) CVI of the entire macular volume scan. To the best of our knowledge, there has not been a head-to-head comparison of 3D and 2D CVI measurements. There is also a need to see whether scanning volume influences 3D CVI measurements.

While it has been reported that 3D CVI of the macula does not exhibit significant topographic variation, it is unknown if this holds true across a range of CT, especially in thicker choroids^18,20,21^. Whether 3D CVI of the macula in eyes with thicker choroids is different has not been investigated. This is important considering the prevalence of the pachychoroid phenotype among Asians^22,23^.

To these ends, we sought to describe the topographic variation of choroidal angioarchitecture of the macula using 3D CVI across a range of CT in healthy eyes from an Asian population and to investigate the associations of CVI.

## Methods

### Study population

The Singapore Epidemiology of Eye Diseases (SEED) Studies comprise prospective population-based cohort studies of Malays, Indians, and Chinese in Singapore^24–27^. The study design has been described in detail previously. The studies are still ongoing. The data for this study was derived from the SEED-2 Study, which is a six-year follow-up study of SEED-1^14^. Stratified sampling by mean subfield subfoveal choroidal thickness (SFCT) was performed by dividing the SFCT data into quintiles and by randomly selecting 10 participants from each quintile. This produced 50 participants who were selected for analysis in this study. This study adhered to the tenets of the Declaration of Helsinki. Ethics approval was obtained from the Institutional Review Board of the Singapore Eye Research Institute. Written informed consent was obtained from all participants.

### Ocular assessment

Each participant underwent a standardised ocular examination by trained operators. Best corrected visual acuity (BCVA) and subjective refraction were measured. Intraocular pressure (IOP) was measured with the Goldmann applanation tonometer (Haag-Streit, Bern, Switzerland) before pupil dilation. AL was measured with a non-contact partial coherence laser interferometer (IOLMaster V.3.01; Carl Zeiss Meditec, Jena, Germany).

### OCT

The Spectralis (Heidelberg Engineering, Heidelberg, Germany) was used to image the choroid under standardised mesopic lighting conditions. After pupil dilation with tropicamide 1% and phenylephrine hydrochloride 2.5%, spectral-domain OCT (SD-OCT) raster scans with enhanced depth imaging (EDI) were acquired on a 30° × 30° macular region centred on the fovea in the High Speed mode, with 31 B-scans per volume scan. Each B-scan was averaged [Automatic Real-time Tracking (ART) mode] using 75 frames, and the distance between consecutive B-scans ranged from 233 to 244 μm. All SD-OCT scans were reviewed by trained graders from the centralised grading centre to ensure that the scans were of sufficient clarity. OCT scans with a signal strength of ≥ 20 were eligible for the analysis. Images with poor focus, motion artifacts, and/or obscure choroid-scleral interface were excluded.

### CVI measurement

To measure CVI, the public domain software ImageJ (Version 1.53c, National Institutes of Health, Bethesda, Maryland, USA)^28,29^ was used to perform image binarisation using techniques that were described by Agrawal et al.^1,2,17^ Image binarisation converts grey scale images into binarised images. An appropriate image binarisation technique is essential to accurately apply a threshold to an image and takes into account illumination, contrast, and resolution of the image pixels.

For 2D CVI (Subfoveal), the foveal OCT B-scan was used. Total choroidal area (TCA) was selected using the polygon tool with the upper border at the RPE-Bruch’s membrane complex and the lower border at the choroid-scleral interface, and added into the Region of Interest (ROI) Manager. Niblack’s auto local thresholding was applied after image conversion into eight bits. This generated the mean pixel value with standard deviation for all points. Image conversion to the Red, Green and Blue (RGB) format was performed to allow the colour threshold tool to select the dark pixels. The TCA and the area of dark pixels, which corresponded to the luminal area (LA), were calculated. To determine the LA within the selected polygon, both the areas in ROI Manager were selected and merged by the “AND” operation of ImageJ. The composite third area was added to the ROI Manager. The first area represents the TCA selected, and the third composite area is the LA. 2D CVI (Subfoveal) was then calculated by dividing LA by the TCA.

**Figure 1:**
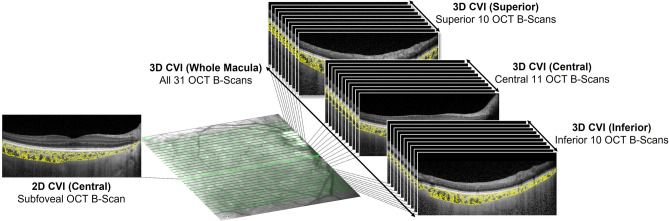
Pictorial representation of the locations in the macula where the various CVI measurements were taken. For 2D CVI (Subfoveal), the foveal OCT B-scan was used. For 3D CVI (Whole Macula), all 31 OCT B-scans were used. The superior 10 OCT B-scans, central 11 OCT B-scans, and inferior 10 OCT B-scans were used for 3D CVI (Superior), 3D CVI (Central), and 3D CVI (inferior), respectively.

3D CVI was subsequently calculated by integrating the 2D CVI across the scan volume. TCA and LA were measured for every OCT B-scan in the volume scan which comprised 31 B-scans. Essentially, choroidal volume (be it the total volume, or that of the stromal or luminal components) in mm^3^ is the summation of the mean area in each B-scan in mm^2^ multiplied by the distance between two consecutive B-scans in μm. 3D CVI was calculated by dividing the luminal volume by the total choroidal volume. This was separately applied for the whole macula (all 31 B-scans), superior macula (superior 10 B-scans), central macula (central 11 B-scans), and inferior macula (inferior 10 B-scans). See Fig. **1** for a pictorial representation of the locations in the macula where the various CVI measurements were taken.

The following steps were applied:

1. Area in the n^th^ OCT B-scan (Area_n_) (for both TCA and LA) in pixel^2^ in each B-scan were converted to mm^2^ by multiplying the area by the scaling factors in the x and y axes:
  Area_n_ (in mm^2^) = Area_n_ (in pixel^2^) * scale_(x axis)_ * scale_(y axis)_; where scale_(x axis)_ is the image resolution in mm/pixel in the x axis and scale_(y axis)_ is the image resolution in mm/pixel in the y axis
2. The mean of areas on two consecutive B-scans (AvgArea) was given by:
  AvgArea = (Area_n_ + Area_n+1_)/2
3. The volume between two consecutive B-scans (Vol) was further given by:
  Vol = AvgArea * T; where T is the inter B-scan distance in μm
4. Lastly, the total volume Vol_Total_ was given by:
  Vol_Total_ = summation of all Vol between two consecutive OCT B-scans in the volume scan.

### CT measurement

CT was automatically measured as the intervening distance between the RPE-Bruch’s membrane complex and the choroid-scleral interface. The slab was segmented on every B-scan of the Spectralis volume scan using the manufacturer’s own algorithm, Heyex SP-X version 6.4.8.116 (exclusively available in the specific research; Heidelberg Engineering). The Heyex software generated a regional CT map with a Early Treatment of Diabetic Retinopathy Study (ETDRS) grid that described the mean subfield CT in all nine ETDRS grid subfields from the 31 horizontal raster B-scans. The B-scans were checked for segmentation artifacts and errors. Misalignment was manually corrected as needed.

### Systemic risk factors

Trained interviewers used standardised questionnaires to collect demographic information, medical history, and lifestyle factors^30^.

Body mass index (BMI) was computed by dividing the body weight (kg) by the square of body height (m). Blood pressure was measured with a digital automatic monitor (Dinamap model Pro Series DP110X-RW, 100V2; GE Medical Systems Information Technologies Inc., Milwaukee, Wisconsin, USA) after five minutes of rest with the participants seated. Hypertension was defined as systolic blood pressure (SBP) of ≥ 140 mmHg, diastolic blood pressure (DBP) of ≥ 90 mm Hg, physician-diagnosed hypertension, or a self-reported history of hypertension.

Non-fasting venous blood was collected for glycosylated hemoglobin (HbA1c), lipid levels, including total cholesterol, high-density lipoprotein (HDL), and low-density lipoprotein (LDL), and serum creatinine. Hyperlipidemia was defined as total cholesterol of ≥ 6.2 mmol/L, or a self-reported use of lipid lowering drugs. Diabetes mellitus was defined as a random glucose level of ≥ 11.1 mmol/L, HbA1c of ≥ 6.5%, use of diabetes mellitus medication, or a physician-diagnosed history of diabetes mellitus. Renal function was assessed using estimated glomerular filtration rate (eGFR) from serum creatinine using the Chronic Kidney Disease-Epidemiology Collaboration equation^31,32^. Chronic kidney disease was defined as an eGFR of < 60 ml/min/1.73 m^2^. Cardiovascular disease was based on a self-reported history of myocardial infarction, angina, or stroke.

### Exclusion criteria

Eyes with BCVA of worse than 20/60, evidence of ocular diseases, or history of ocular surgery were excluded. Eyes with other ocular conditions, such as refractive errors, were not excluded if the OCT scan quality was sufficient to be evaluated.

### Statistical analysis

Statistical analyses were performed using R version 4.1.0^33^. Continuous variables were summarised using means, standard deviations (SD), and 95% confidence intervals (CI), and categorical variables were summarised with counts and percentages. Since CVI and SFCT have different measurement units, we used the coefficient of variation (COV) (%) to compare variability between CVI and SFCT. The normality of the data was assessed with the Shapiro–Wilk test. The comparison of ocular and systemic characteristics among groups was performed using analysis of variance for continuous variables and chi-square tests for categorical variables. Univariate linear regression analyses were performed to examine the associations among ocular and systemic factors with SFCT and CVI. Age and factors that were significant in the univariate analyses (P < 0.05) were included in the multivariable linear regression model. Only one eye from each participant was used. Agreement was assessed using intraclass correlation coefficients (ICC) (two-way random effects, absolute agreement, and single rater/measurement)^34^, Bland–Altman analysis, and F-test for equality of variances. For ICC, values less than 0.5 indicate poor reliability, values between 0.5 and 0.75 indicate moderate reliability, values between 0.75 and 0.9 indicate good reliability, and values greater than 0.90 indicate excellent reliability^34^.

## Results

The ocular and systemic characteristics of the 50 participants are described in Table 1. CVI was similar across the SFCT quintiles. Overall, the 3D CVI of the whole macula was 62.92 ± 1.57%. The 3D CVI of the superior, central, and inferior macula were 62.75 ± 1.93%, 63.35 ± 1.72%, and 62.66 ± 1.70%, respectively. The 2D CVI (Subfoveal) was 63.53 ± 2.09%. The mean SFCT was 288.3 ± 85.6 µm. There were no significant differences in any characteristics among the SFCT quintiles except for proportion of smokers (P = 0.037).

**Table 1:** Ocular and Systemic Characteristics of Participants.

		**Overall**	**SFCT Quintile Ranks**					P*
		**(n = 50 eyes)**	**First (n = 10 eyes)**	**Second (n = 10 eyes)**	**Third (n = 10 eyes)**	**Fourth (n = 10 eyes)**	**Fifth (n = 10 eyes)**	
**Ocular**								
SFCT, µm	Mean (SD)	288.3 (85.6)	174.8 (36.7)	239.5 (14.2)	284.8 (15.3)	331.3 (19.3)	411.2 (44.1)	** < 0.001**
3D CVI (Whole Macula), %	Mean (SD)	62.92 (1.57)	63.41 (1.74)	62.62 (1.54)	62.30 (1.64)	63.50 (1.65)	62.78 (1.20)	0.372
3D CVI (Superior), %	Mean (SD)	62.75 (1.93)	62.76 (2.26)	62.41 (1.43)	62.02 (2.20)	63.78 (1.93)	62.80 (1.66)	0.343
3D CVI (Central), %	Mean (SD)	63.35 (1.72)	64.43 (1.76)	63.02 (1.88)	62.90 (1.66)	63.63 (1.46)	62.75 (1.55)	0.166
3D CVI (Inferior), %	Mean (SD)	62.66 (1.70)	62.92 (2.11)	62.45 (1.78)	61.93 (1.58)	63.16 (1.91)	62.82 (0.96)	0.542
2D CVI (Subfoveal), %	Mean (SD)	63.53 (2.09)	64.30 (2.01)	63.83 (2.35)	63.45 (2.13)	63.19 (1.61)	62.88 (2.37)	0.607
SE, D	Mean (SD)	−0.7 (2.3)	−2.2 (3.3)	−0.3 (1.9)	−0.7 (2.4)	−0.8 (1.9)	0.5 (0.9)	0.119
AL, mm	Mean (SD)	23.9 (1.1)	24.6 (1.5)	23.7 (1.1)	23.8 (0.8)	23.9 (1.3)	23.5 (0.4)	0.191
IOP, mmHg	Mean (SD)	15.1 (3.4)	15.2 (1.7)	14.5 (3.8)	16.2 (4.8)	14.9 (3.7)	14.8 (3.0)	0.848
MOPP, mmHg	Mean (SD)	52.3 (8.0)	54.5 (5.6)	54.8 (10.9)	50.9 (8.4)	49.3 (7.3)	51.8 (7.0)	0.493
**Systemic**								
Age, years	Mean (SD)	53.8 (7.5)	58.7 (9.6)	55.7 (7.1)	51.4 (7.6)	51.7 (5.2)	51.3 (5.1)	0.083
Gender	Male	25 (50.0)	7 (70.0)	4 (40.0)	3 (30.0)	5 (50.0)	6 (60.0)	0.406
	Female	25 (50.0)	3 (30.0)	6 (60.0)	7 (70.0)	5 (50.0)	4 (40.0)	
Ethnicity	Chinese	32 (64.0)	9 (90.0)	7 (70.0)	3 (30.0)	7 (70.0)	6 (60.0)	0.190
	Indian	15 (30.0)	0 (0.0)	3 (30.0)	7 (70.0)	1 (10.0)	4 (40.0)	
	Malay	3 (6.0)	1 (10.0)	0 (0.0)	0 (0.0)	2 (20.0)	0 (0.0)	
Hypertension	No	33 (66.0)	4 (40.0)	6 (60.0)	7 (70.0)	9 (90.0)	7 (70.0)	0.208
	Yes	17 (34.0)	6 (60.0)	4 (40.0)	3 (30.0)	1 (10.0)	3 (30.0)	
Systolic BP, mmHg	Mean (SD)	128.5 (19.2)	132.3 (14.4)	130.8 (22.0)	131.6 (26.1)	121.2 (17.0)	126.5 (15.7)	0.691
Diastolic BP, mmHg	Mean (SD)	76.0 (10.8)	79.3 (8.8)	79.6 (15.4)	73.1 (9.8)	72.6 (9.9)	75.5 (8.6)	0.446
MAP, mmHg	Mean (SD)	93.5 (12.6)	97.0 (9.0)	96.7 (16.9)	92.6 (13.8)	88.8 (11.8)	92.5 (10.5)	0.589
Anti-Hypertensive	No	42 (84.0)	6 (60.0)	10 (100.0)	9 (90.0)	9 (90.0)	8 (80.0)	0.144
	Yes	8 (16.0)	4 (40.0)	0 (0.0)	1 (10.0)	1 (10.0)	2 (20.0)	
Diabetes Mellitus	No	46 (92.0)	9 (90.0)	10 (100.0)	8 (80.0)	9 (90.0)	10 (100.0)	0.433
	Yes	4 (8.0)	1 (10.0)	0 (0.0)	2 (20.0)	1 (10.0)	0 (0.0)	
HbA1c, %	Mean (SD)	5.9 (1.0)	6.2 (1.2)	5.7 (0.3)	5.8 (0.7)	6.1 (1.6)	5.7 (0.3)	0.636
Blood Glucose, mmol/L	Mean (SD)	6.1 (2.6)	6.9 (4.6)	5.2 (0.9)	6.0 (1.4)	6.4 (2.8)	6.1 (2.0)	0.705
Hyperlipidaemia	No	35 (70.0)	7 (70.0)	8 (80.0)	8 (80.0)	6 (60.0)	6 (60.0)	0.753
	Yes	15 (30.0)	3 (30.0)	2 (20.0)	2 (20.0)	4 (40.0)	4 (40.0)	
Total Cholesterol, mmol/L	Mean (SD)	5.4 (0.8)	5.1 (0.7)	5.3 (0.4)	5.5 (0.6)	5.8 (0.9)	5.6 (1.1)	0.350
HDL, mmol/L	Mean (SD)	1.2 (0.3)	1.1 (0.3)	1.3 (0.3)	1.1 (0.3)	1.3 (0.4)	1.2 (0.3)	0.620
LDL, mmol/L	Mean (SD)	3.5 (0.8)	3.1 (0.5)	3.4 (0.5)	3.7 (0.7)	3.7 (0.9)	3.5 (1.1)	0.369
BMI, kg/m^2^	Mean (SD)	24.0 (3.6)	25.3 (2.9)	23.3 (3.7)	24.6 (4.0)	22.7 (4.1)	24.1 (3.2)	0.520
Cardiovascular Disease	No	48 (96.0)	9 (90.0)	10 (100.0)	9 (90.0)	10 (100.0)	10 (100.0)	0.537
	Yes	2 (4.0)	1 (10.0)	0 (0.0)	1 (10.0)	0 (0.0)	0 (0.0)	
Chronic Kidney Disease	No	49 (98.0)	9 (90.0)	10 (100.0)	10 (100.0)	10 (100.0)	10 (100.0)	0.395
	Yes	1 (2.0)	1 (10.0)	0 (0.0)	0 (0.0)	0 (0.0)	0 (0.0)	
Smoker	Never/Past	44 (88.0)	9 (90.0)	10 (100.0)	10 (100.0)	9 (90.0)	6 (60.0)	**0.037**
	Current	6 (12.0)	1 (10.0)	0 (0.0)	0 (0.0)	1 (10.0)	4 (40.0)	

SFCT: subfoveal choroid thickness; SD: standard deviation; 3D: three-dimensional; CVI: choroidal vascularity index; 2D: two-dimensional; SE: spherical equivalent: D: diopter; AL: axial length; IOP: intraocular pressure; MOPP: mean ocular perfusion pressure; BP: blood pressure; MAP: mean arterial pressure; HbA1c: glycated haemoglobin; HDL: high-density lipoprotein; LDL: low-density lipoprotein; BMI: body mass index.

* Statistically significant associations (P < 0.05) are highlighted in bold.

Topographically, there were no significant differences among the 3D CVI of the superior, central, and inferior macula (pairwise comparisons P all > 0.05).

The COV of 3D CVI (Whole Macula), 3D CVI (Superior), 3D CVI (Central), 3D CVI (Inferior), and 2D CVI (Subfoveal) were 2.50%, 3.08%, 2.72%, 2.71%, and 3.29%, respectively, which were lower than that of SFCT (29.69%).

The associations of ocular and systemic factors with CVI and SFCT were studied. 3D CVI (Whole Macula) and 2D CVI (Subfoveal) were only significantly associated with each other. They were not significantly associated with SFCT and other factors. See Table 2, and Supplementary Tables 1 and 2. In the multivariable linear regression analysis, only 2D CVI (Subfoveal) remained significantly associated with 3D CVI (Whole Macula) (ß = 0.41, P < 0.001). See Table 2. Similarly, 2D CVI (Subfoveal) was significantly associated with 3D CVI (Whole Macula) (ß = 0.89, P < 0.001), Total Cholesterol (ß = −0.78, P = 0.036), and cardiovascular disease (ß = 3.65, P = 0.014) in the univariate analysis. However, in the multivariable linear regression analysis, only 3D CVI (Whole Macula) remained significantly associated with 2D CVI (Subfoveal) (ß = 0.85, P < 0.001). See Supplementary Table 1.

**Table 2:** Association of Ocular and Systemic Factors with 3D CVI (Whole Macula).

	Univariate			Multivariable^†^		
	Estimate	Std. Error	P*	Estimate	Std. Error	P*
**Ocular**						
2D CVI (Subfoveal), %	0.48	0.08	** < 0.001**	0.41	0.09	** < 0.001**
SFCT, µm	−2.19 × 10^–5^	2.65 × 10^–3^	0.993	–	–	–
SE, D	0.01	0.10	0.890	–	–	–
AL, mm	−0.23	0.20	0.253	–	–	–
IOP, mmHg	−0.03	0.07	0.619	–	–	–
MOPP, mmHG	−2.88 × 10^–3^	0.03	0.919	–	–	–
**Systemic**						
Age, years	−0.04	0.03	0.207	−0.04	0.02	0.081
Gender, Female	−0.28	0.45	0.529	–	–	–
**Ethnicity**						
Chinese	Reference					
Indian	0.44	0.49	0.375	–	–	–
Malay	1.47	0.99	0.145	–	–	–
Hypertension	−0.04	0.47	0.941	–	–	–
Systolic BP, mmHg	−3.51 × 10^–3^	0.01	0.767	–	–	–
Diastolic BP, mmHg	−3.02 × 10^–3^	0.02	0.866	–	–	–
MAP, mmHg	−4.21 × 10^–3^	0.02	0.816	–	–	–
Anti-Hypertensive	0.54	0.61	0.382	–	–	–
Diabetes Mellitus	0.05	0.83	0.955	–	–	–
HbA1c, %	0.07	0.24	0.782	–	–	–
Blood Glucose, mmol/L	0.02	0.09	0.854	–	–	–
Hyperlipidaemia	−0.06	0.49	0.900	–	–	–
Total Cholesterol, mmol/L	−0.56	0.27	**0.045**	−0.08	0.23	0.715
HDL, mmol/L	−0.66	0.69	0.343	–	–	–
LDL, mmol/L	−0.40	0.28	0.160	–	–	–
BMI, kg/m^2^	0.08	0.06	0.200	–	–	–
Cardiovascular Disease	3.11	1.05	**0.005**	1.84	0.96	0.062
Chronic Kidney Disease	−0.92	1.60	0.566	–	–	–
Current Smoker	−0.33	0.69	0.634	–	–	–

SFCT: subfoveal choroid thickness; Std. Error: standard error; 3D: three-dimensional; CVI: choroidal vascularity index; 2D: two-dimensional; SE: spherical equivalent: D: diopter; AL: axial length; IOP: intraocular pressure; MOPP: mean ocular perfusion pressure; BP: blood pressure; MAP: mean arterial pressure; HbA1c: glycated haemoglobin; HDL: high-density lipoprotein; LDL: low-density lipoprotein; BMI: body mass index.

* Statistically significant associations (P < 0.05) are highlighted in bold.

^†^ Model adjusted R-squared: 0.441; P < 0.001.

In contrast, SFCT was significantly associated with SE (ß = 15.32, P = 0.003), AL (ß = −25.25, P = 0.019), age (ß = −3.66, P = 0.024), and current smoking (ß = 96.04, P = 0.009) in the univariate analysis. These factors remained significant in the adjusted multivariable linear regression analysis: AL (ß = −19.97, P = 0.043), age (ß = −3.31, P = 0.025), and current smoking (ß = 82.29, P = 0.015). See Supplementary Table 2. Notably, the adjusted R-squared values of the multivariable analyses were higher for 3D CVI (Whole Macula) and 2D CVI (Subfoveal) at 0.441 and 0.503, respectively, in comparison with that for SFCT at 0.248.

**Figure 2:**
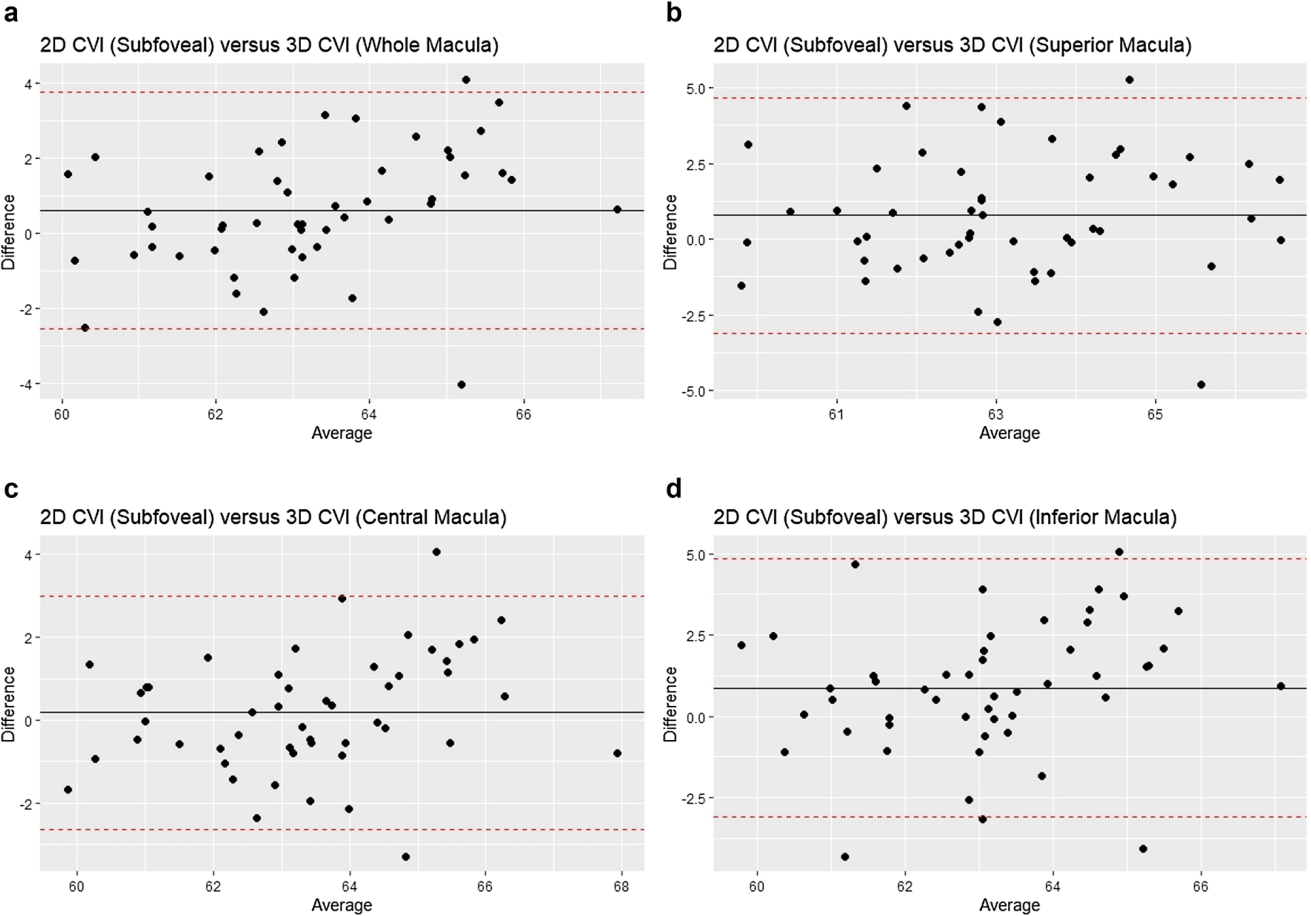
**a** Bland–Altman analysis of 2D CVI (Subfoveal) versus 3D CVI (Whole Macula). This is a plot of the difference [2D CVI (Subfoveal) –- 3D CVI (Whole Macula) versus the mean of the two measurements). **b** Bland–Altman analysis of 2D CVI (Subfoveal) versus 3D CVI (Superior Macula). This is a plot of the difference [2D CVI (Subfoveal)—3D CVI (Superior Macula) versus the mean of the two measurements). **c** Bland–Altman analysis of 2D CVI (Subfoveal) versus 3D CVI (Central Macula). This is a plot of the difference [2D CVI (Subfoveal) – 3D CVI (Central Macula) versus the mean of the two measurements). **d** Bland–Altman analysis of 2D CVI (Subfoveal) versus 3D CVI (Inferior Macula). This is a plot of the difference [2D CVI (Subfoveal) – 3D CVI (Inferior Macula) versus the mean of the two measurements). The solid black line represent the mean differences and the interrupted red lines represent the 95% limits of agreement.

Next, pairwise agreements between 2D CVI of the subfoveal OCT B-scan and the 3D CVI of the macula (Whole Macula, Superior, Central, and Inferior) were assessed. See Table 3. 2D CVI (Subfoveal) had only a moderate agreement with 3D CVI (Central) (ICC = 0.719, 95% CI: 0.554 to 0.830). The agreement of 2D CVI (Subfoveal) with 3D CVI (Whole Macula), 3D CVI (Superior), and 3D CVI (Inferior) were poorer (ICC = 0.591, 95% CI: 0.365 to 0.749; ICC = 0.483, 95% CI: 0.234 to 0.671; and ICC = 0.394, 95% CI: 0.134 to 0.604, respectively). The Bland–Altman analysis showed a mean difference of 0.18% between 2D CVI (Subfoveal) and 3D CVI (Central) and a 95% limit of agreement of between −2.63% to 3.00%. The mean differences between 2D CVI (Subfoveal) and 3D CVI (Whole), 3D CVI (Superior), and 3D CVI (Inferior) were larger and ranged from 0.61% to 0.81%, and the 95% limits of agreement were wider. See Fig. 2.There was also an indication that in the pairwise comparison between 2D CVI (Subfoveal) and 3D CVI (Whole Macula), the proportionate difference [given by 2D CVI (Subfoveal)—3D CVI (Whole Macula)] increased as the 2D CVI (Subfoveal) increased. See Fig. 2a. There were no significant differences in variances except for a marginal difference between 2D CVI (Subfoveal) and 3D CVI (Whole Macula), P = 0.049.

**Table 3:** Agreement between 2D CVI (Subfoveal) and 3D CVI (Whole, Superior, Central, and Inferior Macula).

	ICC (95% CI)	Bland–Altman		F-test in equality of variance (P)*
		95% Limits of agreement, %	Mean difference, % (95% CI)	
2D (Subfoveal) versus 3D (Whole Macula)	0.591 (0.365, 0.749)	−2.55, 3.76	0.61 (0.15, 1.07)	**0.049**
2D (Subfoveal) versus 3D (Superior Macula)	0.483 (0.234, 0.671)	−3.11, 4.66	0.78 (0.21, 1.34	0.600
2D (Subfoveal) versus 3D (Central Macula)	0.719 (0.554, 0.830)	−2.63, 3.00	0.18 (−0.22, 0.59)	0.179
2D (Subfoveal) versus 3D (Inferior Macula)	0.394 (0.134, 0.604)	−3.10, 4.85	0.87 (0.30, 1.45)	0.153

ICC: intraclass correlation coefficient: CI: confidence interval. * Statistically significant associations (P < 0.05) are highlighted in bold.

**Figure 3:**
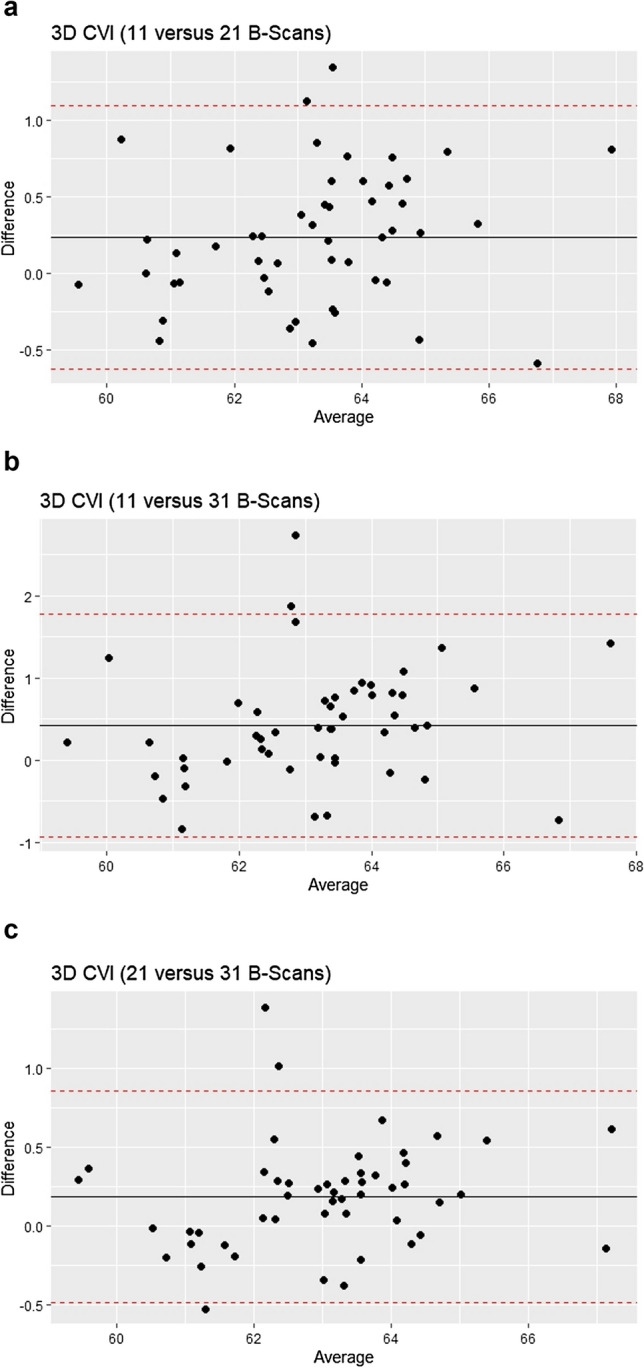
**a** Bland–Altman analysis of 3D CVI (11 B-Scans) versus 3D CVI (21 B-scans). This is a plot of the difference [3D CVI (11 B-Scans)—3D CVI (21 B-Scans) versus the mean of the two measurements). **b** Bland–Altman analysis of 3D CVI (11 B-Scans) versus 3D CVI (31 B-scans). This is a plot of the difference [3D CVI (11 B-Scans)—3D CVI (31 B-Scans) versus the mean of the two measurements). **c** Bland–Altman analysis of 3D CVI (21 B-Scans) versus 3D CVI (31 B-scans). This is a plot of the difference [3D CVI (21 B-Scans)—3D CVI (31 B-Scans) versus the mean of the two measurements). The solid black lines represent the mean differences and the interrupted red lines represent the 95% limits of agreement.

Lastly, the influence of scanning volumes on CVI was assessed. See Supplementary Fig. 1 for a pictorial representation of the regions in the macula that were compared. The ICC, Bland–Altman analysis, and F-test for equality of variances are shown in Table 4. The 3D CVI measurements showed good to excellent agreement regardless of the number of B-scans used from the volume scan. The ICC was more than 0.85 for all three comparisons (11 versus 21 B-scans: ICC = 0.957, 95% CI: 0.897 to 0.979; 11 versus all 31 B-scans: ICC = 0.884, 95% CI: 0.712 to 0.945; and 21 versus all 31 B-scans: ICC = 0.971, 95% CI: 0.930 to 0.986). The Bland–Altman analysis showed low mean differences (range: 0.19% to 0.24% and narrow 95% limits of agreement. See Fig. 3. There were no significant differences in variances for all three comparisons among 11, 21, and all 31 B-scans. However, the agreement was still better for 21 versus 31 B-scans compared with 11 versus 21 B-scans (higher ICC, narrower 95% limits of agreement, smaller mean difference, and smaller difference in variance).

**Table 4:** Relationship between Scanning Volume and 3D CVI Measurements.

	ICC (95% CI)	Bland–Altman		F-test in equality of variance (P)
		95% Limits of agreement, %	Mean difference, % (95% CI)	
11 versus 21 B-Scans	0.957 (0.897, 0.979)	−0.62, 1.10	0.24 (0.11, 0.36)	0.725
11 versus 31 B-Scans	0.884 (0.712, 0.945)	−0.93, 1.78	0.42 (0.23, 0.62)	0.529
21 versus 31 B-Scans	0.971 (0.930, 0.986)	−0.49, 0.86	0.19 (0.09, 0.28)	0.781

ICC: intraclass correlation coefficient: CI: confidence interval.

## Discussion

This study has described the topographic variation of choroidal angioarchitecture of the macula using 3D modelling of the choroid in healthy eyes from an Asian population and investigated the associations of CVI. The novel aspects of this study are the evaluation of the topographic variation of 3D CVI across a range of SFCT and the head-to-head comparison of 3D and 2D CVI measurements in the posterior pole of healthy eyes.

3D CVI demonstrated little topographic variation across the macula regardless of SFCT. The 3D CVI of the superior, central, and inferior regions of the macula were similar. This is consistent with the results of previous studies. Agrawal et al. highlighted that a single foveal scan is representative of the whole posterior pole choroidal vascularity in healthy participants^18^. Goud et al. reported no significant differences in CVI among different sectors of the ETDRS grid^20^. Similarly, Breher et al. reported that CVI was consistent across the central 10 mm of the retina^21^. Notably these studies had fewer participants, did not examine the topographic variation of 3D CVI across a range of CT, and used automatic analysis of the OCT volume scans.

Similar to that reported in previous studies^1,2,17^, our results indicate that CVI was resistant to the influence of ocular and systemic factors. 3D and 2D CVI demonstrated less variability than SFCT. On multivariable linear regression, 3D CVI (Whole Macula) and 2D CVI (Subfoveal) were only significantly associated with each other. They were not significantly associated with SFCT and the other ocular and systemic factors like SE, AL, mean ocular perfusion pressure (MOPP), age, ethnicity, and mean arterial pressure. These results agree with the existing literature. In a study of healthy participants by Agrawal et al., CVI was not significantly associated with ocular factors including AL, IOP, and mean ocular perfusion pressure, and systemic factors including age, gender, BMI, systolic and diastolic BP, serum glucose, HbA1c, cholesterol, blood creatinine, current smoking, and alcohol consumption^17^. A separate study by Singh et al. which used wide-field OCT also showed that gender, refraction, IOP, and AL did not have significant associations with CVI^35^.

Notably, the adjusted R-squared values of the multivariable analyses were higher for 3D CVI (Whole Macula) and 2D CVI (Subfoveal), compared with that for SFCT. This indicated that the assessed factors accounted for more of the variation for CVI compared with SFCT.

Both 3D and 2D CVI were not significantly related to age. This agrees with previous studies by Agrawal et al.^17^ and Oh et al. on Asian participants^36^, and also by Zhou et al. on a Caucasian population^19^. In contrast, Ruiz-Medrano et al. described that CVI was significantly higher in participants under 18 years of age compared with older participants and attributed it to a decrease of the LA with age while the SA remained stable^37^. Nivison-Smith et al. also described that CVI significantly decreased from the age of 33 to 43 years at a rate of 0.7–2.7% per decade^2^. Inconsistencies in results may be due to differences in methods, including imaging algorithms, binarisation techniques, OCT devices and imaging modality, assessed regions, and inter-participant differences^19^.

To the best of our knowledge, there has not been a head-to-head comparison of 3D and 2D CVI measurements in the posterior pole of healthy eyes. 2D CVI (Subfoveal) had only a moderate agreement with 3D CVI in the central macular region of 11 B-scans. The agreement of 2D CVI (Subfoveal) with 3D CVI (Whole Macula), 3D CVI (Superior), and 3D CVI (Inferior) were poorer. The Bland–Altman analysis showed a similar picture, with a smaller mean difference and a narrower 95% limit of agreement for 2D CVI (Subfoveal) versus 3D CVI (Central), compared with 2D CVI (Subfoveal) versus 3D CVI of the whole, superior, and inferior macula.

In this regard, 2D CVI of the fovea is only partially representative of 3D CVI of the central macula in healthy eyes. Our results reflect differences in 2D and 3D CVI measurements. The latter involve incorporation of inter-scan distances and scaling factors in the x and y axes. Also, that the 2D CVI (Subfoveal) showed a better agreement with the 3D CVI of the central macula region as opposed to that of the whole macula and of the other regions reflects subtle topographic differences across the macula. Therefore, CVI of a single 2D B-scan may not be a representative indicator of the choroidal vasculature. The choroid vascular status should be assessed using 3D information of the choroid rather than using a 2D scan. The assessment of 3D CVI may be particularly useful in understanding the changes in the overall and regional choroidal vasculature in diseases involving part of the choroid or of the entire choroid, e.g., age-related macular degeneration, diabetic retinopathy, inherited retinal diseases, posterior uveitis, etc. The regional topographical variations in the 3D CVI can potentially allow novel pathomechanisms of the disease, and accordingly, selective pharmacotherapy, to be identified.

There was also an indication that in the pairwise comparison between 2D CVI (Subfoveal) and 3D CVI (Whole Macula), the proportionate difference [given by 2D CVI (Subfoveal)—3D CVI (Whole Macula)] increased as the 2D CVI (Subfoveal) increased. This is an interesting observation. For choroids with a greater CVI, one should be mindful that 3D CVI measurements of the whole macula may diverge from 2D CVI of the subfoveal region.

Lastly, the comparison of 11, 21, and 31 B-scans showed that there was no significant influence of the scanning volume on the 3D CVI measurements of the macula. The ICC was more than 0.85 (good to excellent) for all three comparisons. The Bland–Altman analysis showed low mean differences and narrow 95% limits of agreement. Reducing the number of OCT B-scans is appealing to increase cost-effectiveness and increase patient compliance during the OCT scan.

However, the agreement was still better for 21 versus 31 B-scans compared with 11 versus 21 B-scans. It is logical that measuring 3D CVI over a large macular area will produce measurements closer to the true measurement. This is also more essential in diseased eyes if it has a localised pattern. Nonetheless, this comes at the expense of a longer scanning time and more effort being spent on data and image processing.

The key strength of this study lies in the assessment of 3D CVI from the entire volume scan, which is a more representative and relevant approach for the assessment of the choroidal vasculature and architecture, as compared with an assessment of a single subfoveal scan or of the average of selected B-scans across the macula. The novel analyses include the head-to-head comparison of 2D and 3D CVI measurements and the assessment of scanning volume on 3D CVI measurements. The data set was obtained via stratified sampling from the SFCT quintiles of a population-based study and has a wide representation of CT profiles. Standardised clinical examination protocols were used in our study.

This study has limitations. Firstly, a limited number of healthy eyes were studied, and even fewer in each SFCT quintile. The study is underpowered to perform comparisons of 3D CVI among the SFCT quintiles and among the macular regions. We were not able to perform sample size calculations a priori for this study. Using the means and standard deviations of the differences in 3D CVI (Whole Macula) among the different SFCT quintiles, approximately 100 eyes in each quintile will be required to achieve a power of 80% and a level of significance of 5% in two-sided tests. Nonetheless, we hope that this will help guide sample size considerations in future studies. Secondly, the topographic variation and the associations of 3D CVI may not apply to diseased eyes, especially if the disease process is localised. It will be interesting to assess 3D CVI in diseased eyes. Thirdly, the CVI measurements were time-consuming and laborious. Therefore, clinical utility may be limited. For 50 participants with 31 B-scans in each volume scan, a total of 1550 B-scans were manually segmented. By applying automated algorithms, these processes can be performed automatically and expediently. Zhou et al. have described that automated assessments of the choroid have good agreement with manual segmentations^19^.

In summary, this study has described the topographic variation of choroidal angioarchitecture and associations of 3D CVI in healthy eyes. CVI was not correlated with demographic or ocular structural features. 2D CVI of the fovea is partially representative of 3D CVI of the macula in healthy eyes, thus the choroid vascular status should be assessed using 3D information of the choroid rather than by a 2D scan. Lastly, there is no significant influence of scanning volume on 3D CVI measurements of the macula.

## Supplementary Information

Supplementary Information 1.

Supplementary Information 2.
