# Supplementary figures and images for: Three-dimensional modelling of the choroidal angioarchitecture in a multi-ethnic Asian population
Source: Sci Rep. 2022 Mar 9;12:3831. doi: 10.1038/s41598-022-07510-y (PMC8907174; doi:10.1038/s41598-022-07510-y)

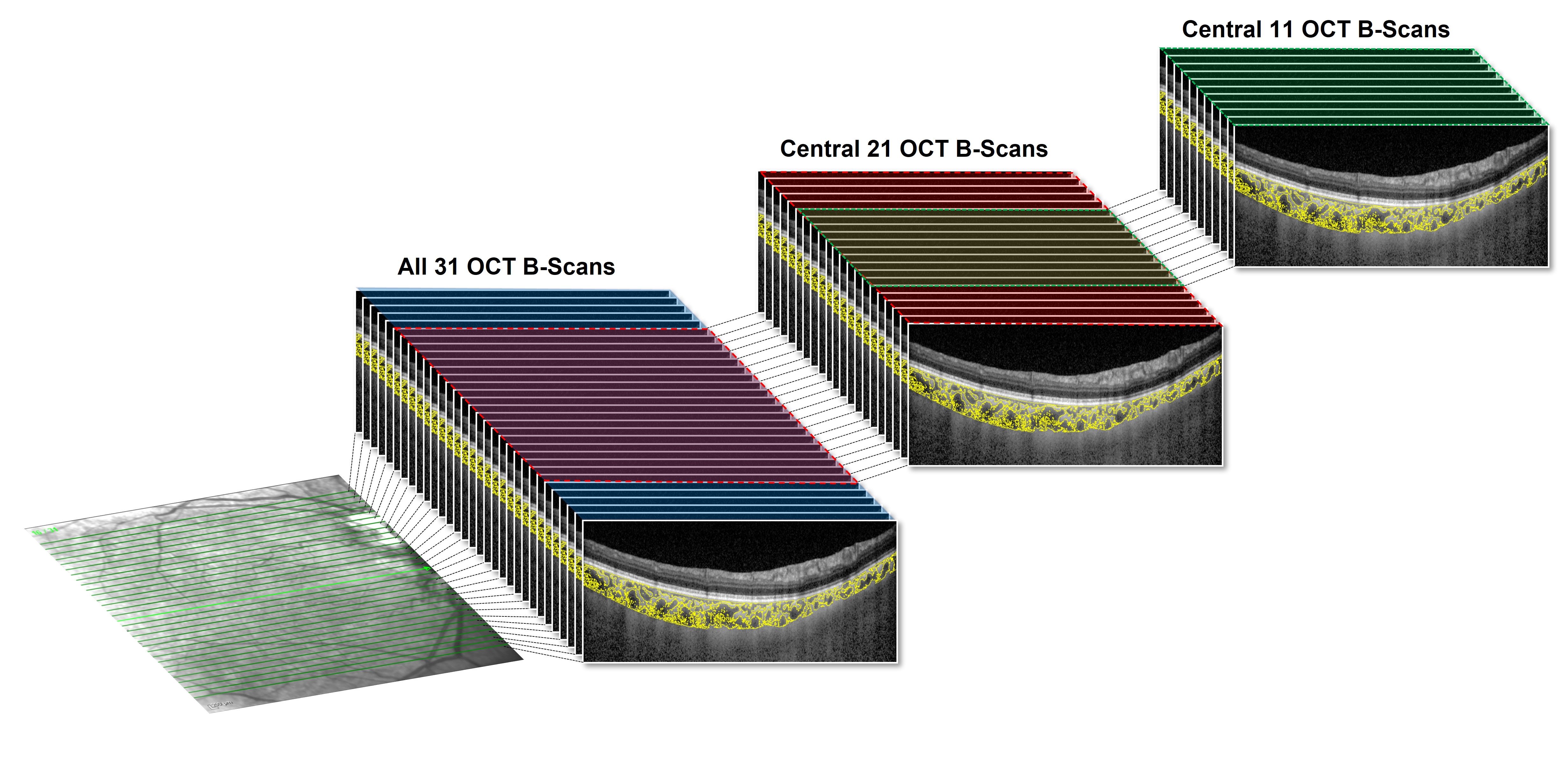

Supplement: Supplementary file 1 — Supplementary Information 1. [file 41598_2022_7510_MOESM1_ESM.jpg]
