# Supplementary material for: Three-dimensional modelling of the choroidal angioarchitecture in a multi-ethnic Asian population
Source: Sci Rep. 2022 Mar 9;12:3831. doi: 10.1038/s41598-022-07510-y (PMC8907174; doi:10.1038/s41598-022-07510-y)
Supplement: Supplementary file 2 — Supplementary Information 2. [file 41598_2022_7510_MOESM2_ESM.docx]

**Supplementary Information**

**Three-dimensional modelling of the choroidal angioarchitecture in a multi-ethnic Asian population**

Kai Xiong Cheong,^1^ Kelvin Yi Chong Teo,^1-2^ Yih Chung Tham,^1-2^ Ralene Sim,^1^ Shivani Majithia,^1^ Jia Min Lee,^3^ Anna Cheng Sim Tan,^1-2^ Ching-Yu Cheng,^1-2^ Chui Ming Gemmy Cheung,^1-2^ Rupesh Agrawal^3-5*^

^1^ Singapore Eye Research Institute, Singapore National Eye Centre, Singapore

^2^ Ophthalmology & Visual Sciences Academic Clinical Program (Eye ACP), Duke-NUS Medical School, Singapore

^3^ National Healthcare Group Eye Institute, Tan Tock Seng Hospital, Singapore, Singapore

^4^ Lee Kong Chian School of Medicine, Singapore

^5^ Moorfields Eye Hospital, NHS Foundation Trust, London, United Kingdom

* Corresponding Author

**Correspondence and reprint requests to:**

Associate Professor Rupesh Agrawal

National Healthcare Group Eye Institute, Tan Tock Seng Hospital

11 Jalan Tan Tock Seng, Singapore 308433

Phone: (+65) 63577726 Fax: (+65) 63577718

Email: rupeshttsh@gmail.com

**Legends**

**Supplementary Figure 1.** Pictorial representation of the regions in the macula that were compared to assess the influence of scanning volumes on 3D CVI measurements in the macula. The green, red, and blue sectors comprise the central 11 OCT B-scans, central 21 OCT B-scans, and all 31 OCT-B scans in the scan volume, respectively.

**
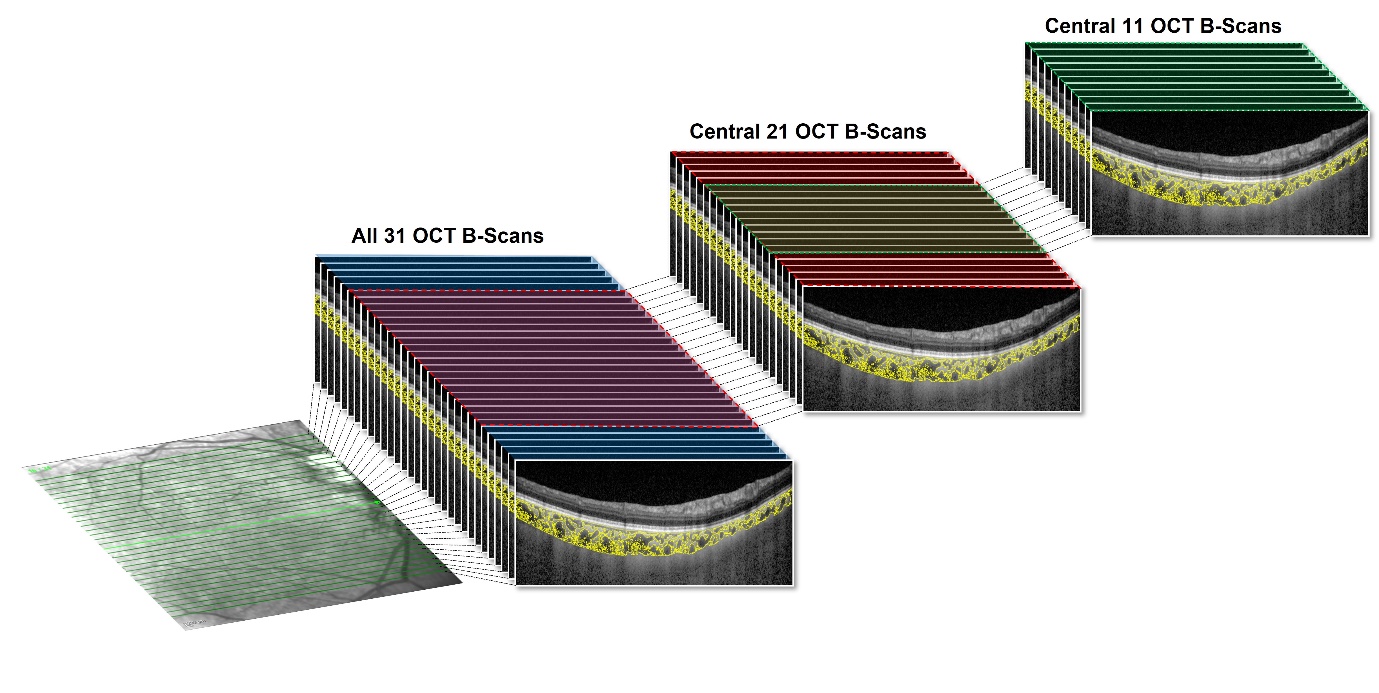
**

**Supplementary Table 1: Association of Ocular and Systemic Factors with 2D CVI (Subfoveal)**

|  | **Univariate** | | | **Multivariable^†^** | | |
| --- | --- | --- | --- | --- | --- | --- |
|  | **Estimate** | **Std. Error** | **P*** | **Estimate** | **Std. Error** | **P*** |
| **Ocular** |  |  |  |  |  |  |
| 3D CVI (Whole Macula), % | 0.89 | 0.12 | **<0.001** | 0.85 | 0.14 | **<0.001** |
| SFCT, µm | -4.99x10^-3^ | 3.44x10^-3^ | 0.153 | - | - | - |
| SE, D | -0.11 | 0.13 | 0.396 | - | - | - |
| AL, mm | -0.20 | 0.27 | 0.455 | - | - | - |
| IOP, mmHg | -0.06 | 0.08 | 0.500 | - | - | - |
| MOPP, mmHG | 0.02 | 0.04 | 0.676 | - | - | - |
| **Systemic** |  |  |  |  |  |  |
| Age, years | -0.02 | 0.04 | 0.699 | 0.01 | 0.03 | 0.729 |
| Gender, Female | 0.77 | 0.59 | 0.192 | - | - | - |
| Ethnicity |  |  |  |  |  |  |
| Chinese | Reference |  |  |  |  |  |
| Indian | 0.68 | 0.65 | 0.304 | - | - | - |
| Malay | 1.55 | 1.32 | 0.248 | - | - | - |
| Hypertension | 0.51 | 0.62 | 0.420 | - | - | - |
| Systolic BP, mmHg | 7.45x10^-3^ | 0.02 | 0.636 | - | - | - |
| Diastolic BP, mmHg | -1.28x10^-3^ | 0.03 | 0.964 | - | - | - |
| MAP, mmHg | 0.01 | 0.02 | 0.830 | - | - | - |
| Anti-Hypertensive | 0.61 | 0.81 | 0.451 | - | - | - |
| Diabetes Mellitus | 1.06 | 1.09 | 0.336 | - | - | - |
| HbA1c, % | 0.35 | 0.31 | 0.270 | - | - | - |
| Blood Glucose, mmol/L | 0.16 | 0.11 | 0.157 | - | - | - |
| Hyperlipidaemia | 0.27 | 0.65 | 0.685 | - | - | - |
| Total Cholesterol, mmol/L | -0.78 | 0.36 | **0.036** | -0.11 | 0.29 | 0.707 |
| HDL, mmol/L | 0.24 | 0.93 | 0.795 | - | - | - |
| LDL, mmol/L | -0.60 | 0.37 | 0.114 | - | - | - |
| BMI, kg/m^2^ | 0.04 | 0.08 | 0.612 | - | - | - |
| Cardiovascular Disease | 3.65 | 1.43 | **0.014** | 0.63 | 1.23 | 0.610 |
| Chronic Kidney Disease | -1.43 | 2.12 | 0.504 | - | - | - |
| Current Smoker | -1.31 | 0.90 | 0.152 | - | - | - |

SFCT: subfoveal choroid thickness; Std. Error: standard error; 3D: three-dimensional; CVI: choroidal vascularity index; 2D: two-dimensional; SE: spherical equivalent: D: diopter; AL: axial length; IOP: intraocular pressure; MOPP: mean ocular perfusion pressure; BP: blood pressure; MAP: mean arterial pressure; HbA1c: glycated haemoglobin; HDL: high-density lipoprotein; LDL: low-density lipoprotein; BMI: body mass index

* Statistically significant associations (P < 0.05) are highlighted in bold.

^†^ Model adjusted R-squared: 0.503; P<0.001

**Supplementary Table 2: Association of Ocular and Systemic Factors with SFCT**

|  | **Univariate** | | | **Multivariable^†^** | | |
| --- | --- | --- | --- | --- | --- | --- |
|  | **Estimate** | **Std. Error** | **P*** | **Estimate** | **Std. Error** | **P*** |
| **Ocular** |  |  |  |  |  |  |
| 3D CVI (Whole Macula), % | -0.07 | 7.87 | 0.993 | - | - | - |
| 2D CVI (Subfoveal), % | -8.41 | 5.80 | 0.153 | - | - | - |
| SE, D | 15.32 | 4.90 | **0.003** | - | - | - |
| AL, mm | -25.25 | 10.41 | **0.019** | -19.97 | 9.60 | **0.043** |
| IOP, mmHg | -1.88 | 3.58 | 0.603 | - | - | - |
| MOPP, mmHG | -1.99 | 1.52 | 0.196 | - | - | - |
| **Systemic** |  |  |  |  |  |  |
| Age, years | -3.66 | 1.57 | **0.024** | -3.31 | 1.43 | **0.025** |
| Gender, Female | 6.48 | 24.45 | 0.792 | - | - | - |
| Ethnicity |  |  |  |  |  |  |
| Chinese | Reference |  |  |  |  |  |
| Indian | -23.03 | 27.15 | 0.401 | - | - | - |
| Malay | -14.60 | 54.87 | 0.791 | - | - | - |
| Hypertension | -50.84 | 24.76 | 0.455 | - | - | - |
| Systolic BP, mmHg | -0.65 | 0.64 | 0.312 | - | - | - |
| Diastolic BP, mmHg | -1.72 | 1.12 | 0.130 | - | - | - |
| MAP, mmHg | -1.35 | 0.96 | 0.167 | - | - | - |
| Anti-Hypertensive | -19.87 | 33.25 | 0.553 | - | - | - |
| Diabetes Mellitus | -30.24 | 44.88 | 0.504 | - | - | - |
| HbA1c, % | -13.05 | 12.85 | 0.315 | - | - | - |
| Blood Glucose, mmol/L | -2.55 | 4.69 | 0.590 | - | - | - |
| Hyperlipidaemia | 29.45 | 26.35 | 0.269 | - | - | - |
| Total Cholesterol, mmol/L | 25.28 | 15.11 | 0.101 | - | - | - |
| HDL, mmol/L | 28.67 | 37.85 | 0.453 | - | - | - |
| LDL, mmol/L | 18.46 | 15.46 | 0.238 | - | - | - |
| BMI, kg/m^2^ | -4.46 | 3.39 | 0.195 | - | - | - |
| Cardiovascular Disease | -32.63 | 62.25 | 0.603 | - | - | - |
| Chronic Kidney Disease | -95.22 | 86.29 | 0.275 | - | - | - |
| Current Smoker | 96.04 | 35.00 | **0.009** | 82.29 | 2.73 | **0.015** |

SFCT: subfoveal choroid thickness; Std. Error: standard error; 3D: three-dimensional; CVI: choroidal vascularity index; 2D: two-dimensional; SE: spherical equivalent: D: diopter; AL: axial length; IOP: intraocular pressure; MOPP: mean ocular perfusion pressure; BP: blood pressure; MAP: mean arterial pressure; HbA1c: glycated haemoglobin; HDL: high-density lipoprotein; LDL: low-density lipoprotein; BMI: body mass index

* Statistically significant associations (P < 0.05) are highlighted in bold.

^†^ Model adjusted R-squared: 0.248; P=0.001
